# Cognitive representation of gait: differences in memory structures between individuals after total knee arthroplasty and total hip arthroplasty

**DOI:** 10.1007/s10339-024-01255-4

**Published:** 2025-01-27

**Authors:** Dagmar Linnhoff, René Kaiser, Klaus Mattes, Cornelia Frank

**Affiliations:** 1 Department of Human Movement Science, University of Hamburg, Turmweg 2, 20148 Hamburg, Germany; 2 Department of Clinical Research and Sport Science, OrthoCentrum Hamburg, Hansastr. 1—3, 20149 Hamburg, Germany; 3 Human Movement Science Group, University of Bremen, Am Fallturm 1, 28359 Bremen, Germany

**Keywords:** Structural dimensional analysis, Mental representation, Gait deviation, Functional representation structure, Long-term memory, Arthroplasty

## Abstract

The objective was to examine differences in the gait-specific cognitive representation structures between individuals after total knee- (TKA) and after total hip-joint arthroplasty (THA). The cognitive representation structure was compared between three groups: 1. three months after TKA (*n* = 12), 2. three months after THA (*n* = 12), and 3. healthy control group (CG) (*n* = 12) using the structural dimensional analysis of mental representation (SDA-M). Additionally, perceived joint function was rated by either the KOOS, JR. or HOOS, JR. Mean distribution of perceived joint function was not significantly different between the TKA (60.35 ± 11.2) and THA group (68.01 ± 13.8) (t = − 1.425; *p* = .173). In the cognitive representation structure, the THA group exhibited functional differences from the TKA group and control group, both of which showed a functional structure. Three months after hip joint replacement the gait-specific cognitive representation structure seems to reflect joint function-specific deviations. Therefore, focussing on functional recovery of cognitive gait representation may facilitate gait rehabilitation in individuals after hip replacement.

## Introduction

Osteoarthritis is considered the most common joint disease worldwide and most often affects the knee joint, followed by the wrist and the hip (Hunter and Bierma-Zeinstra 2019; Steinmetz et al. 2023). The course of the disease is associated with pain and restrictions in daily life situations of affected persons (Neogi 2013; Hunter et al. 2014). Restrictions in walking, as they occur with severe arthrosis of the knee or hip joint, severely limit mobility and thus social participation, especially among older individuals (Hefter and Götz 2013). In this case, knee or hip replacement surgery is commonly recommended if appropriate conventional therapies have been unsuccessful (Hunter and Bierma-Zeinstra 2019; Carr 2012). The lifetime risk of total hip replacement (THA) at the age of 50 years has been estimated at 11.6% for women and 7.1% for men, and for knee replacement (TKA), 10.8% and 8.1% for women and men respectively (Culliford et al. 2012). Knee or hip replacement surgery aims to restore the physiological basis for unrestricted walking by replacing the affected joint with an artificial joint (Brocks 2007). Despite this restoration, walking after surgery must be relearned because physiological structures must regenerate and the gait pattern must be adapted to the new joint (Cassel et al. 2017; Heisel and Jerosch 2007). Thus, gait rehabilitation is essential for patients in the first weeks after TKA or THA (Bandholm et al. 2018; Jöllenbeck and Pietschmann 2019; Snell et al. 2018).

Despite participation in gait rehabilitation programs, research shows that functional postoperative outcomes and daily activity levels after TKA or THA often do not return to their pre-pathology level (Luna et al. 2017; Lyman et al.; 2020; Smith et al. 2015). Regarding pre-operative gait, studies found that although the gait pattern improves significantly in the first few weeks after surgery, it still differs from that of non-affected persons after a year or more. Usually, healthy or normal gait is associated with gait symmetry which means that the left and right leg are relatively equal in terms of spatio-temporal and dynamic gait parameters (Viteckova et al. 2018). Deviations or asymmetries that occur as a result of pain avoidance (e.g. unequal weight distribution or stance time) are often found in arthritic gait but also still after joint replacement, leading to an overuse of other structures (Jöllenbeck and Pietschmann 2019). For individuals after TKA, Perka et al. (2000) found that gait symmetry improved 28 weeks after surgery compared to before, but still deviated from the normal range found in unrestricted gait. Berth et al. (2002) found that the range of motion of the knee was still significantly lower for individuals 33 months after TKA compared to a control group. Also, Yoshida et al. (2012) reported differences in kinematic, kinetic, and spatiotemporal variables compared to individuals without knee pathology three years post-surgery. In the case of THA, Leuchte et al. (2007) reported that asymmetric loading rates remained at 28 weeks after surgery. Ten months after THA, Beaulieu et al. (2010) found that the sagittal-plane range of motion was still lower in the operated joint compared to the non-operated. In accordance, Foucher et al. (2007) found that despite good subjective functional rating, the range of motion in the sagittal plane and the internal rotation moments of the hip joint one year after THA still differed from those of a control group with radiographically normal hips. Overall, the reported studies show that some gait deficits persist after joint replacement of either the knee- or the hip joint and thus can lead to long-term overuse of healthy structures, limited function and activity. This raises the question of what causes gait deviations remaining after joint replacement and physical rehabilitation.

Motor behaviour such as gait depends on physiological structures, but also and in particular relies on the individuals’ movement-related cognitive-perceptual memory structures (Bernshteĭn 1967; Schack 2004, 2020). According to the authors, movement execution is controlled by the cognitive representation of the (expected) perceptual effects. This cognitive-perceptual perspective on motor control postulates that cognitive and motor processes underlying action control are functionally linked. Following this line of reasoning, the cause of the remaining gait deficits may be cognitive in nature. Replacing a joint with an artificial one changes the individual’s perception of the joint (Collins 2012). The cognitive-perceptual memory structures would need to adapt to the change by integrating the sensory information perceived by the new joint. Thus, it can be assumed that both, the physiological state and the adaptation of gait-specific cognitive structures, influence the postoperative gait pattern. To date, not much is known about cognitive-perceptual memory structures after joint replacement surgery. Considering that gait performance is influenced by representational networks stored in long-term memory, it may be helpful to assess these gait-related, cognitive representations when developing indication-specific, effective rehabilitation strategies for individuals to overcome the aforementioned persisted gait deviations.

The cognitive action architecture approach (Schack 2004, 2020) is based on this cognitive-perceptual view on movement control. It says that procedural knowledge is hierarchically organized and basic action concepts (BACs) serve as cognitive representation units between the action goal (top) and the motor commands (bottom). These can be understood as representational units in long-term memory (LTM) that integrate the functional and sensory features of movements. The way that movement-related BACs are structurally organized or clustered is crucial for movement execution and can be accessed with specific psychometric procedures (e.g. structural dimensional analysis) (Schack 2012, 2020). Using these procedures, research has indicated that the hypothesized relationship between clustering of movement-related BACs in LTM and movement execution exists in different movement contexts like tennis, golf, judo, volleyball, dancing, or manual action (Bläsing et al. 2009; Gromeyer et al. 2022; Land et al. 2013; Stöckel et al. 2012; Velentzas, 2011; Weigelt et al. 2011). Frank et al. (2013) additionally showed in a pre-post design that the structure of mental representation changes during the motor learning process towards a more functional clustering. Last, the structuring or clustering of BACs in the cognitive representation can directly be related to movement execution errors (Schack and Mechsner 2006).

Exploring the relation between the gait-specific cognitive representation and disordered gait has received growing interest in research to date. Jacksteit et al. (2018) compared the mental representation of gait between persons suffering from severe knee arthrosis and same-aged healthy persons. They identified seven BACs representing the structure of a single gait cycle (foot strike to foot strike of one leg). The authors found structural differences between both groups in that the control group exhibited a representation structure that separated the BACs into two functional phases while in the knee-arthrosis group two crucial sub-phases of gait (mid stance and mid swing) were separated from the remaining BACs. From these results, the authors concluded that in healthy individuals the two functional phases represent the functionally correct deviation into a stance- and swing phase according to the literature (Perry and Burnfield 2010). In contrast, the cognitive representation of persons with severe knee arthrosis exhibited either a dysfunction of weight-bearing on the affected leg (mid stance) or swinging the affected leg forward (mid swing). In another setting, Jacksteit et al. (2019) extended their results from patients with knee- to patients with hip arthrosis in conducting a three group cross-sectional study (1. persons with severe hip-osteoarthritis, 2. persons six months after total hip arthroplasty (THA) surgery, and 3. Same-aged healthy controls). They found similar results for the comparison between the control group and the hip-arthrosis group as former for the knee-arthrosis group compared to the control group. Here, the hip-arthrosis group also functionally separated mid stance and mid swing from the remaining BACs whereas the control group did not. However, in individuals six months after hip replacement surgery, the mid swing phase was functionally integrated while the mid stance was still functionally separated. The authors concluded that six months after THA, the mental representation is more functional than it is pre-surgery but is still different from undisturbed gait. Finally, in a longitudinal study, Schega et al. (2014) investigated whether an intervention with visual feedback leads to changes in gait-specific cognitive representation of individuals shortly after THA. In a randomized controlled trial, they measured the gait-specific mental representation of 20 persons who underwent THA 14 days before and assigned them either to a visual feedback intervention group or to a gait intervention control group (receiving no visual feedback). Statistical comparison of the individual representation structures with a given functional reference (as a measure of representation quality) revealed that the two groups did not differ before the intervention, whereas the group who received visual feedback was significantly closer to the reference after the intervention. The authors concluded that the visual feedback intervention led to an improvement in gait-specific cognitive representation structure.

In summary, the following conclusions can be deduced from the findings: First, the gait-related cognitive representation structure in individuals with hip- and knee arthrosis (i.e. before joint replacement) differs from healthy persons (Jacksteit et al. 2018; Jacksteit et al. 2019). For either group (knee- and hip-arthrosis) in the distinct studies, this deviation from healthy individuals was explained by the fact that they experienced pain and gait limitations through their condition. Second, six months after hip joint replacement, the gait-specific cognitive representation of patients differs both from healthy persons and also from individuals suffering from hip arthrosis. At this point, no comparable study exists for individuals after knee-joint replacement leaving open the question of whether this is also true after knee joint replacement. From a cognitive-perceptual view, a new artificial joint brings along new joint-specific perceptual input affecting the cognitive representation of gait. Therefore, with regard on the gait-specific representation structure in LTM, both joint replacement groups should differ from each other and also from unimpaired individuals. If this is the case, it would indicate that the former mentioned persisted gait deviations are (at least partly) of cognitive origin embedded in LTM. For rehabilitation practice this would indicate to not only focus on physiological but also joint function specific training integrating exercises that target weak points indicated by the cognitive representation structure. Second, the monitoring of therapy success may be extended by accessing the cognitive representation structure. The aim of this study was to explore whether the expected differences in the gait-specific representation can be empirically proven, leading to the former mentioned indications for gait rehabilitation.

## Methods

### Ethics statement and informed consent

This cross-sectional study was carried out in the Biomechanics Laboratory of the Hamburg University and the OrthoCentrum Hamburg. The study was approved by the Ethical Review Committee of the Department of Psychology and Movement Science of the Hamburg University (No. 2020_330) and performed in accordance to their guidelines as well as in accordance to the Declaration of Helsinki (2013) and the DFG (German Research Foundation) regulations for Good Research Practice. Informed consent was obtained from all participants prior to inclusion into the study.

### Population

Groups were defined as (1) persons three months after total knee arthroplasty (TKA), (2) persons three months after total hip arthroplasty (THA), and (3) same-aged healthy persons (control).

To draw conclusions on the question of whether the mental representation structure of individuals after knee joint replacement functionally differs from those after hip joint replacement, cognitive representation structures should ideally be measured when patients have overcome physical limitations caused by surgery. This is when patients are admitted to full-weight bearing and getting back to their preoperative activity status at 8 to 12 weeks after surgery (Lyman et al. 2020; Ribeiro-Castro 2024).

Participants after THA or TKA were recruited from the OrthoCentrum in Hamburg, which is an orthopaedic clinic that specialized in knee and hip surgery. They qualified for participation if they underwent surgery 90 ± 10 days ago following a diagnosis of degenerative arthrosis and were allowed to use full weight-bearing. Furthermore, the medical records indicated that no surgery-related complications had occurred, that they had no injuries or limitations in the untreated leg and no limitations other than treated osteoarthritis in the operated leg, and that no cognitive limitations had been diagnosed. The criteria were checked against the medical staff in the medical records and patients were asked if there had been any leg injuries or walking limitations in the last five years. Participants after TKA or THA were finally included after consultation with their attending doctor. Healthy controls were recruited via advertisements. They qualified for participation if they were between 50 and 80 years old, had no history of lower extremity surgery or lower extremity injury, and had not been diagnosed with any neurological conditions that affected their gait or any cognitive limitations. They were asked for their health status and possible exclusion criteria prior to participation. Comparable studies that have measured the mental representation of patients with gait disorders used sample sizes of 10 to 20 participants per group (Jacksteit et al. 2018; Jacksteit et al. 2019; Schega et al. 2014). Accordingly, the total sample size was set at *N* = 36 (mean age = 66.25, ± 8.4; 27 females) with 12 participants per group (TKA: mean age = 67.42 ± 9.7, 9 females; THA: mean age = 65.17 ± 9.9, 8 females; control: mean age = 66.17 ± 5.7, 9 females). The time after surgery was 89.42 ± 3.2 days for those in the TKA and 86.83 ± 6.7 days for those in the THA group, and the right leg was affected in 75% of cases (see Table 1 for details).

**Table 1:** Study population details mean ± SD

Group (m/f)	n	Age	Days after surgery	Score KOOS/HOOS	Left/right leg
TKA (3/9)	12	67.42 ± 9,7	89.42 ± 3.2	60.35 ± 11.2	3/9
THA (4/8)	12	65.17 ± 9.9	86.83 ± 6.7	68.01 ± 13.8	4/8
Control (3/9)	12	66.17 ± 5.7	–	–	–

### Measuring mental representation

We used structural dimensional analysis of mental representation (SDA-M) which is a psychometric method to access the structure of movement representation in long-term memory (Schack 2012). This method aims to reveal the hierarchical arrangement of basic action concepts (BACs) in long-term memory by a decision task (splitting procedure) performed by the participant. The decisions concern the functional affiliation of the BACs.

**Fig. 1:**
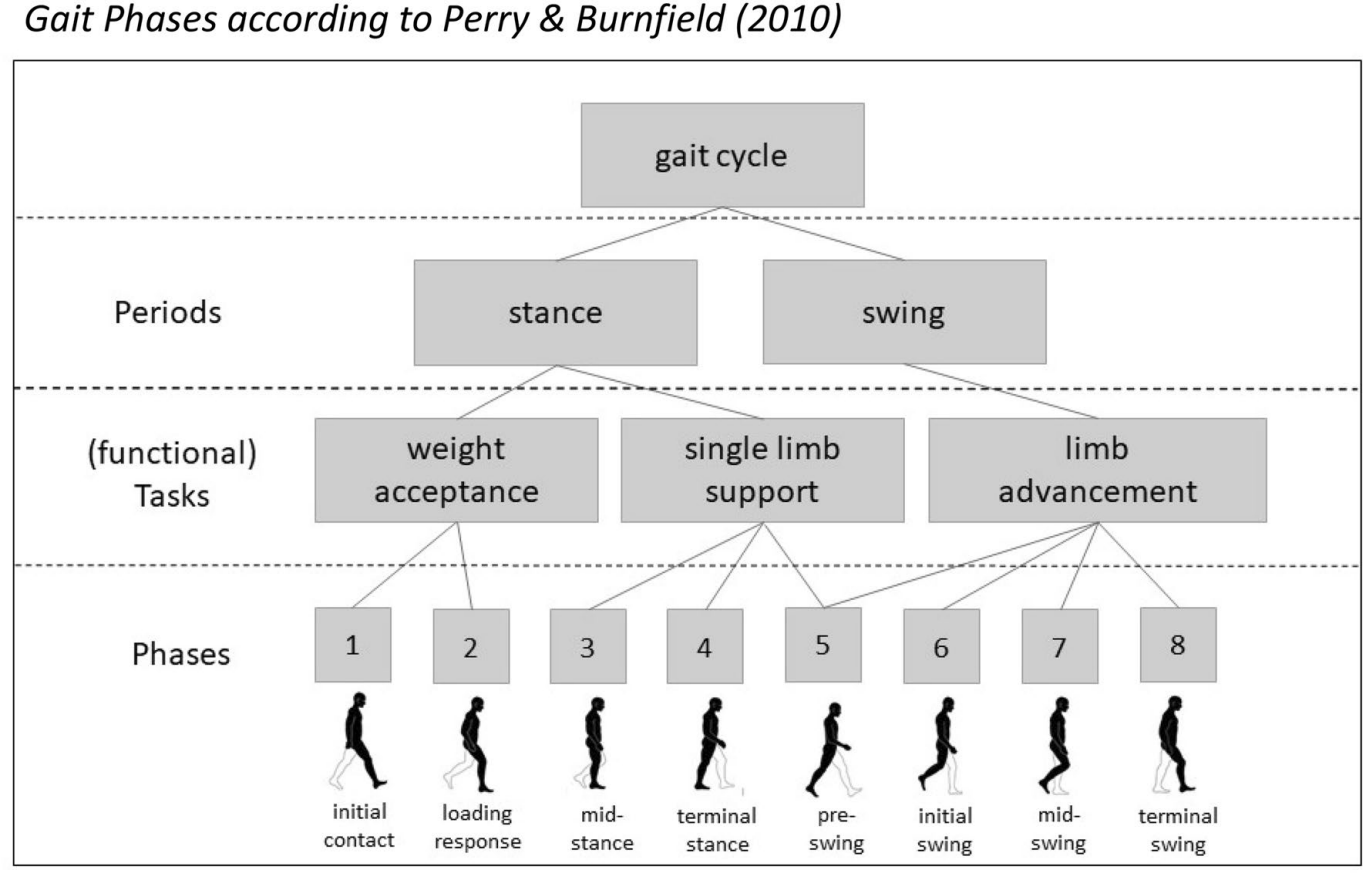
*Gait Phases according to *Perry and Burnfield (2010)

For the purpose of the present study, we determined gait-specific BACs according to the gait phases suggested by Perry and Burnfield (2010). The model proposes eight successive gait phases, which are assigned to two main phases (1. stance and 2. swing) and three main functional tasks within the gait cycle (1. weight acceptance, 2. single limb support, 3. limb advancement) (Fig. 1). For each of the eight functional phases, a short and intuitive item was phrased in German language, resulting in a final set of 8 BACs (Table 2).

**Table 2:** *BACs of gait based on *Perry and Burnfield (2010)

BAC Number	Phase	German language	English translation
1	Initial contact	Der Fuß setzt vor dem Körper auf dem Boden auf	The foot touches down on the ground in front of the body
2	Loading response	Das Körpergewicht wird auf den Fuß übernommen	The body weight is transferred to the foot
3	Mid stance	Das Körpergewicht wird auf den vorderen Teil des Fußes verlagert	The body weight is transferred to the front part of the foot
4	Terminal stance	Die Ferse löst sich vom Boden	The heel lifts off the ground
5	Pre swing	Der Fuß wird entlastet	The foot bears less weight
6	Initial swing	Der Fuß verlässt den Boden	The foot leaves the ground
7	Mid swing	Der Fuß schwingt nach vorn	The foot swings forward
8	Terminal swing	Der Fuß bewegt zum Boden	The foot moves towards the ground

*The BACs phrased in German language were used in the study. The BACs in English were added for better readability*

With the final set of 8 BACs (Table 2), the SDA-M was set up for the study using the QSplit software version 1.6 (Bielefeld University, Neurocognition and Action Group) that contains all functions and algorithms needed for data analysis.

To determine distances between BACs, a splitting task is used followed by mathematical statistical procedures on the obtained data (Lander 1991; Schack 2012). In the splitting task, for each possible combination of two BACs, participants must decide whether two given BACs are related during movement execution or not (for more details on the splitting procedure, see Procedure). The starting point for all further analysis is the Z-transformation of this decision data (related/not related) for each BAC into a Z-matrix, which opens up two analysis options: (1) obtaining and visualizing the representation structure via hierarchical cluster analysis (CA), and (2) statistically comparing obtained cluster solutions by determining their invariance (Schack 2012).

(1) To obtain and visualize the hierarchical representation structure the Z-matrix is transformed into a Euclidian distance (d) matrix and hierarchical cluster analysis (CA) is performed. The result of the CA is a dendrogram showing individually build clusters of BACs and the Euclidian distances (d) as hierarchical order criterion. All intersections that are below the critical d-value (d_krit_) (based on the selected α) are statistically clustered in the individual representation. Individual cluster solutions (dendrograms) can also be summed up to form a group structure.

(2) The comparison of individual representation structures is based on invariance analysis (between two cluster solutions/dendrograms), which can be performed using an invariance measure (λ) that reflects the pairwise congruencies between two cluster solutions (Lander 1991; Schack 2012).

### Procedure

Before participation, written informed consent was obtained from all participants. To assess the perceived functional status in the two post-surgery groups before testing either the short version of the Knee Injury and Osteoarthritis Outcome (KOOS, JR.) or the Hip Injury and Osteoarthritis Outcome (HOOS, JR.) was administered to the TKA and THA group. The KOOS, JR. is a seven-item patient-reported outcome measure (PROM) representing “knee health” as a one-dimensional construct (Lyman et al. 2016a). The HOOS, JR. consists of six items representing the dimension “hip health” (Lyman et al. 2016b). The patient-reported outcome measures (PROMs) were used to control for similarity of perceived rehabilitation status among both groups. Since the total score of each questionnaire is translated into a scale from 0 to 100% function, their scores are directly comparable.

The splitting procedure was performed using a standard laptop (Samsung Series 5 Ultrabook) running the Q-Split software (version. 1.6) and displayed using a standard computer monitor (Hanns.G, Hi221D).

**Fig. 2:**
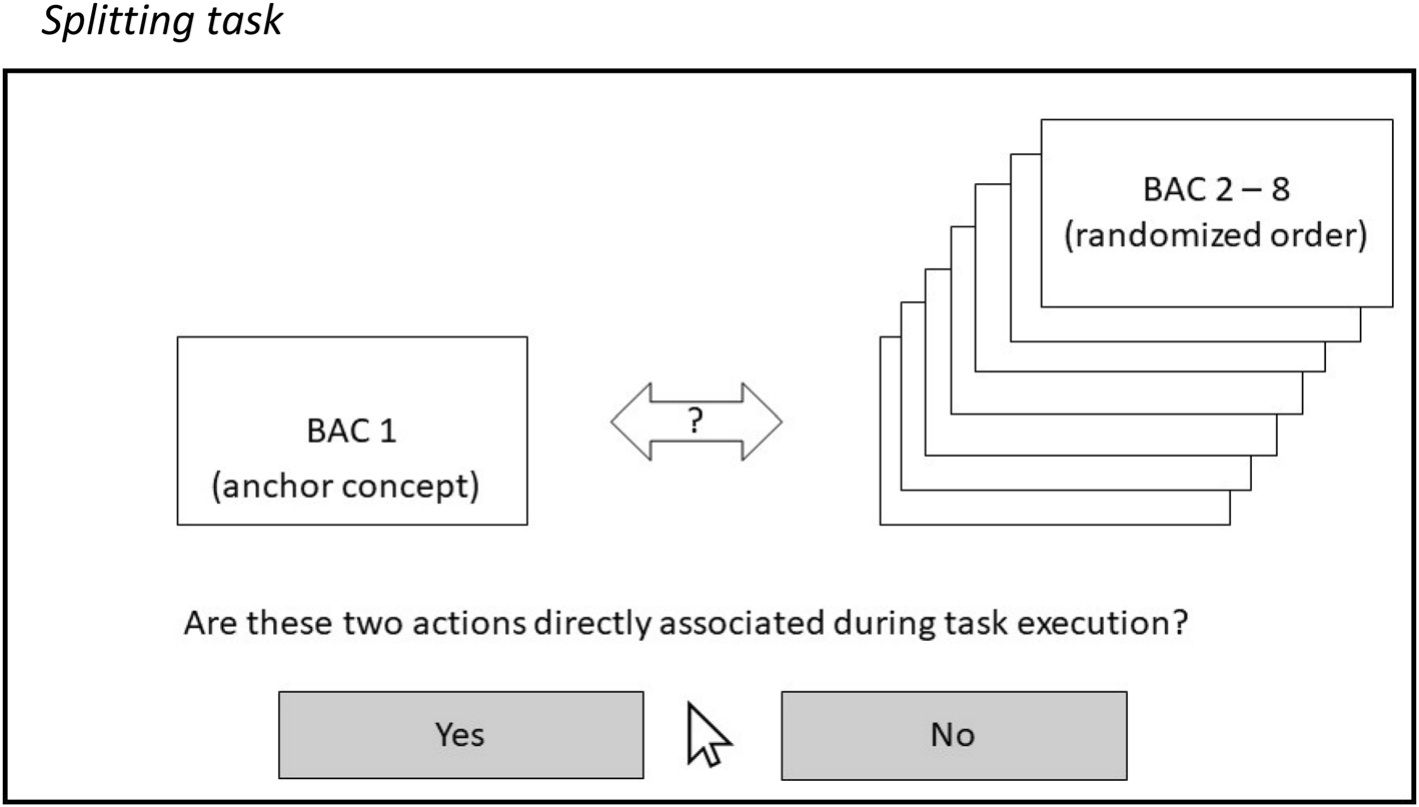
Splitting task. Note: schematic illustration of the splitting procedure as it appears on the screen. One BAC serves as anchor concept (left side) and is compared to the remaining seven BACs (right side)

The splitting task proceeds as follows: A selected BAC is permanently displayed on the left side of the screen (anchor concept), while the remaining (seven) BACs are presented on the right side one after the other in randomized order. For each paring, participants then have to decide whether the two currently presented BACs are related or not during movement execution. Subsequently, the next BAC serves as the anchor concept and the procedure continues. The splitting task ends after each BAC is compared to all remaining BACs in the list. Accordingly, 56 (8 × 7) decisions were made by mouse click for "yes" or "no" (Fig. 2). Participants were instructed by using a standardized PowerPoint presentation to ensure that it was the same instruction in each case. In the instruction, participants were asked to decide intuitively whether or not the two movements (displayed BACs) were directly related during movement execution (of gait). Additionally, participants in the TKA and THA groups were instructed that each movement description referred to their affected leg, while the control group was told that the movements always referred to the same leg. No other decision criterion was given. Before the start of the measurement, participants were asked to perform three trial decisions (using the same program) to ensure that they understood the task and were comfortable with the procedure.

### Data processing and analysis

Psychometric data on the cognitive representation structure was obtained from each individual after finishing the splitting task. The statistical procedures were carried out as described above: group dendrograms for the three distinct groups (1. TKA, 2. THA, 3. control) were obtained using the Q-Split software implemented grouping function. This function is based on hierarchical cluster analysis to reveal which BACs are significantly related throughout the group. A critical distance value of d_krit_ = 3.53, reflecting an alpha level of α = 0.05, was used to determine statistically relevant links between BACs. This means that BACs that are interconnected above a critical distance value (d_krit_) of 3.53 are statistically not related (Schack et al. 2012). Additionally, the statistical invariance between the three group dendrograms was determined. As a statistical measure of structural invariance, λ-values for each paring (TKA vs. THA; TKA vs. control; THA vs. control) were calculated by way of analysis of invariance of the clusters of each paring (λ) (Lander et al. 1991). According to the literature, λ < 0.68 (which reflects the top two interval criteria) reveals that two cluster solutions are significantly different at an alpha level of α = 0.05 (Lander et al. 1991; Schack et al. 2012).

As a metric measure of how strongly BACs of one functional task are associated, we calculated an average distance of pairwise distances between BACs per functional task (function-specific distance). For this purpose, we manually retrieved all pairwise distances between BACs belonging to one of the three functional tasks, i.e., weight acceptance (BAC 1–2), single limb support (BAC 3–5) and limb advancement (BAC 5–7). As suggested by Jacksteit et al. (2018, 2019), we did not include BAC 8 into this calculation because BAC 8 overlaps with BAC 1 due to the cyclic nature of gait. Finally, we compared the function-specific distances between all three groups via ANOVA and Bonferroni corrected post hoc tests for each functional task.

## Results

With regard to the perceived functional status (KOOS, JR or HOOS, JR) a mean score of 68.0 for the THA and 60.4 for the TKA group were found respectively. Statistical analysis of the mean distribution between groups confirmed that both groups were not significantly different from each other (t = − 1.425; *p* = 0.173) in terms of their perceived functional status of the affected joint.

**Fig. 3:**
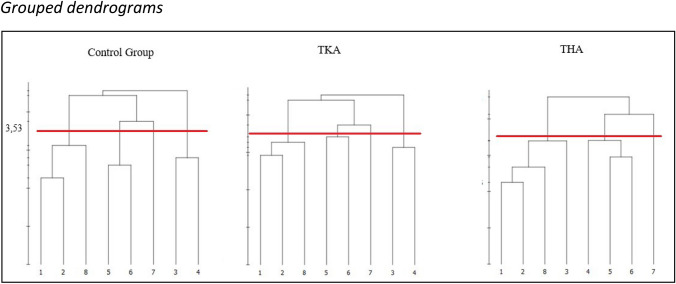
Grouped dendrograms. Note: Mean group dendrograms for (left) age-matched control group, (middle) total knee arthroplasty group, (right) total hip arthroplasty group. The y-axis displays the distances between the basic action concepts (BACs). The red line marks the critical distance (d_krit_)of 3,53, below which the links between the BACs are considered statistically related. The numbers indicate the BACs: (1) initial contact, (2) loading response, (3) mid-stance, (4) terminal stance, (5) pre-swing, (6) initial swing, (7) mid-swing, (8) terminal swing

Structural comparison of group dendrograms revealed differences between the two groups of patients (TKA and THA) in terms of functional clustering of the gait-related sub-phases or BACs (Fig. 3). Two independent functional phases could be identified for the THA group that included seven of the eight given BACs (BAC 1,2,3,8 and BAC 4,5,6). The first functional phase included the sub-phases: initial contact (1), loading response (2), mid stance (3), and terminal swing (8). The second phase included the sub-phases: terminal stance (4), pre swing (5), and initial swing (6). In contrast, three functional phases could be identified for the TKA group including the same seven BACs but differently clustered (BAC 1,2,8, BAC 3,4, and BAC 5,6). Here the first phase included: initial contact (1), loading response (2), and terminal swing (8). The second phase included the sub-phases: mid stance (3) and terminal stance (4) and the third phase included the sub-phases: pre swing (5) and initial swing (6). For the control group, the clustering of BACs was equal to the clustering of the TKA group. The sub-phase mid-swing (BAC 7) was statistically not included in any functional cluster in all three groups.

Analyses of invariance revealed that the cluster solutions of the TKA- and THA group were significantly different (i.e., variant) from each other (λ = 0.52). The THA group was also significantly different from the control group (λ = 0.52). The comparison between the control group and the TKA group revealed no difference (i.e., invariance) in their representation structures (λ = 1.00).

The respective function-specific distances for each functional task can be seen in Table 3. For weight acceptance, there was no significant difference between the three groups (*F* = 0.970; *p* = 0.390; η^*2*^ = 0.06). For single limb support, there was a significant difference between the three groups (*F* = 3.729; *p* = 0.035; η^*2*^ = 0.18). Post hoc tests revealed that the difference was significant between the THA and the control group (*p* = 0.043) and between THA and TKA (*p* = 0.043). For limb support, there was also no significant difference between all three groups (*F* = 0.297; *p* = 0.745; η^*2*^ = 0.02).

**Table 3:** Average pairwise distances per functional task

	Weight acceptance (BAC 1–2)	Single limb support (BAC 3–4)	Limb advancement (BAC 5–7)
Control	2.49 ± 0.89	2.90 ± 0.94	3.35 ± 0.64
TKA	3.10 ± 0.79	3.11 ± 0.86	3.56 ± 0.86
THA	2.65 ± 1.49	3.91 ± 1.07	3.53 ± 0.61

Function-specific distances (Mean ± SD) calculated for the three functional gait tasks for the three groups: Control group, patients after total knee replacement (TKA), patients after total hip replacement (THA)

## Discussion

Since not much is known about joint-function related differences in the cognitive representation structure present in individuals after joint replacement, we compared the gait-specific cognitive representation structures between individuals three months after total knee replacement surgery (TKA), individuals three months after total hip replacement surgery (THA) and healthy same-aged persons. By using structural-dimensional analysis of mental representations (SDA-M), it was possible to reveal the cognitive representation of gait, and as such to determine the structuring of related action concepts stored in long-term memory in TKA and THA patients. We hypothesized that individuals would differ with respect to their gait-specific cognitive representational structure. We expected the gait-specific cognitive representation structure of both joint replacement groups to be less functionally organized compared to not-impaired, same-aged persons. Further, we expected both joint replacement groups to deviate in their representation structure with regard to the function of the affected joint. By this comparison, we aimed to investigate whether the gait-specific cognitive representation structure (stored in the LTM) reflects the joint-specific impairments on gait.

The first main finding was, that the grouping of BACs in the same aged healthy control group corresponded to the three functional tasks of gait, namely weight acceptance, single limb support, and limb advancement as suggested by Perry and Burnfield (2010) (see Fig. 1). This was also supported by the function-specific distances computed for the respective functional tasks, which were lowest in this group. Related studies also found that healthy individuals exhibited a gait-specific cognitive representation structure that corresponded to functional gait phases as defined in the model (Jacksteit 2018, 2019; Schega et al. 2014; Stöckel et al. 2015). We thus replicate the findings from Jacksteit et al. (2018, 2019) and Stöckel (2015) by showing that individuals with unimpaired gait hold functional cognitive representation structures of gait in their long-term memory.

As an extension, and in line with our predictions, the gait-specific cognitive representation structure of the THA group was different from the TKA group and also from the control group as revealed by invariance analysis. Further, the assumption that their representation structure would deviate in a function-specific way was supported by the results. The THA group revealed a representation structure clustered into two main phases, with mid stance (BAC 3) and terminal stance (BAC4) being seperated (Fig. 2). According to the model structure (Perry and Burnfield 2010), both sub-phases belong to the stance phase and share the main functional task of single limp support. This functional deviation was also supported by comparing the function-specific distances between the groups, as only the THA group deviated from the other two groups in this functional phase. In other words, the last part of the stance phase was misallocated into the swing phase. In fact, this functional misallocation might reflect a difficulty in the transition from stance to swing within the gait cycle. From a movement execution perspective, this is when the leg is being extended at the hip joint and therefore can be assumed to be a hip-joint function specific deviation. In this case, it can be followed that the gait-specific structure of mental representation for the THA group seems to reflect a joint-specific problem. This is in line with previous findings (Jacksteit et al. 2019; Schega et al. 2014).

Looking more closely at the functional clustering of BACs, there are differences between this study and the studies by Jacksteit et al. (2018, 2019). Notably, both used the same approach to access the structure cognitive representation. In the case of individuals after THA, Jacksteit et al. (2019) also showed two functional clusters but not build of the same BACs. In both studies however, a part of the stance-phase was integrated with the swing-phase. For same-aged healthy controls, Jacksteit et al. (2018, 2019) found a representation structure similar to that we found in the THA group. Both deviations may be due to the fact that although we used the same approach, there may have been differences in instructions given to the participants.

In contrast to our predictions, the representation structure of the TKA group was statistically indifferent to that of the control group. The representation of joint specific problems expected for the TKA group was not reflected in their cognitive representation structure. A possible explanation could be that the cognitive representation structure in the TKA group three months after surgery may already reflect an advanced approximation towards a functional structure. Compared to the control group, the TKA group showed the same functional clusters but higher function-specific distances in all three functional tasks, indicating that their representation structure was less elaborated. It may be that the predicted function-specific differences may have been present earlier after surgery, while having disappeared after three months of recovery. Currently, there are no other studies available that investigated the gait-specific cognitive representation structure in individuals after total knee arthroplasty.

The fact that Jacksteit et al. (2018) found differences between individuals with severe knee osteoarthritis and healthy controls indicates that their gait-specific cognitive representation structure differs before surgery. Our results raise the question of why the cognitive representation structure of the TKA group might recover faster than it does in the case of the THA group. One explanation, stemming from a functional, cognitive-perceptual perspective, may be that the function of the knee joint within the gait cycle might be less complex due to its fewer degrees of freedom. More research is needed to explain differences in adaptation and change in their cognitive-perceptual memory structures.

In summary, three months after joint replacement surgery, participants after total knee replacement showed a gait-specific cognitive representation structure that is very close to that of healthy individuals, while this was not the case for individuals after hip joint replacement. Both joint replacement groups did not significantly differ from each other in their perception of the functional status of the affected joint (as indicated by the questionnaires). Rather, the THA group tended to perceive the functional status of the joint better than the TKA group did (based on the mean values of the KOOS, JR., and HOOS, JR. scores). This trend is supported by studies that found that after the same time of rehabilitation, perceived functional status is better in individuals after hip replacement than after knee replacement (Beer et al. 2012; Collins et al. 2012). Thus, the fact that the THA group exhibited limitation-specific changes in cognitive representation, whereas the TKA group did not, is an unexpected and interesting finding. In turn, this would imply that the cognitive representational structure reveals limitations that are not reflected in the subjective assessment of joint function. This finding underlines the assumption of an unconscious nature of knowledge that is functionally relevant for the control and organization of actions as described in the cognitive architecture model (Schack 2012, 2020). Evidence was found to support the hypothesis that the robust gait disturbances found in people who have undergone knee or hip replacement are related to the representation of movement in long-term memory. Therefore, examination of cognitive representation structures, in addition to clinical assessments and gait measurements, has the potential to provide additional valuable insight helping to understand, support and monitor gait rehabilitation after joint replacement.

Along these lines, the Correct Action Sequence Probability Analysis (CASPA) (Strenge et al. 2019, 2020) may be an adequate means for error prediction in future studies and clinical application in gait rehabilitation. This analysis extends the SDA-M to sequential action, and allows for prediction of errors that may occur in a given action sequence. Specifically, the probability of the occurrence of a sequential action execution error can be estimated based on the obtained distance data. An important prerequisite for the application of CASPA is, however, that the respective task is sequential in nature. Since gait is not strictly sequential due to its cyclic nature, and since BACs of the current set do overlap (in sequential terms) due to shared functional characteristics, one should carefully consider how to adapt the set of BACs accordingly if used for gait. Specifically, future studies with a focus on execution errors in gait sequences such as dysfunctional walking after surgery may adapt the set of BACs towards a strictly sequential one, e.g., by avoiding overlap such as start and end of gait (BAC 8) as suggested by Jacksteit et al. 2018, 2019. Taking these necessary conditions into account, this opens up perspectives for future research on the relation between movement execution and cognitive representation and provides a means to develop targeted gait assistance and training tailored to individual prerequisites in gait therapy.

### Limitations

Since this was a cross-sectional study design, it is not possible to draw conclusions about changes in representations, and how the structure of cognitive representation has developed from surgery up to that point in time after some time of rehabilitation. It would be interesting to know whether both groups of patients differed from each other before surgery and whether long-term memory structures have changed from before to after surgery in each group. Here, another important link for future research becomes apparent, in that change in cognitive-perceptual memory structures over time should be investigated in more detail. In order to examine the role that the gait-specific cognitive representation plays in long-term gait rehabilitation after joint replacement, research looking at changes in representation structures by way of a longitudinal design is particularly needed.

To deeper evaluate the effect of cognitive memory structures on gait behaviour, measuring biomechanical gait parameters, such as joint kinematics, could be performed in addition to that of the cognitive representation structure. Drawing conclusions about relationships between biomechanical parameters and mental representation can be helpful in understanding how the representation structure influences movement execution (Land et al. 2013; Schack 2012).

The requirement for the validity of our conclusions was that both patient groups were comparable. The criterion of elapsed time after surgery was also used in many other comparative studies between knee- and hip-joint replacement (Bandholm et al. 2018; Beer et al. 2012; Collins et al. 2012; Lyman et al. 2020; Snell et al. 2018,). We additionally collected data on subjectively perceived joint function in order to rule out the possibility that both groups differed significantly in their state of recovery. It should be noted that although a direct comparison of the two joint-specific questionnaires (KOOS, JR. and HOOS, JR.) is common in orthopaedic research, comparing two different joints is only possible to a limited extent. For future studies, it would be recommended to use the same clinical assessment in all groups. Nevertheless, it must be acknowledged that all possible confounders cannot be excluded in this design and controlling as many as possible is important.

Last, we retrieved information on the cognitive function of the participants via medical records and from themselves but did not screen the participants with validated clinical assessments. Since the splitting procedure as main part of our analysis is sensitive to the participants understanding of the task, ensuring normal cognitive function in a population of higher age is recommended for future research.

## Conclusion

This study has shown that the cognitive-perceptual representation structure of individuals three months after total hip replacement deferred from the structure found in individuals three months after total knee replacement and from individuals with undisturbed gait. This indicates that long-term gait disturbances go along with a function-specific altered cognitive representation in case of individuals three months after total hip replacement. The gait-specific mental representation structure of this group revealed that the transition from stand to swing phase was not represented functionally correctly. For gait therapy in individuals after total hip arthroplasty, this implies that when reaching a plateau in progression after 12 weeks, focusing on the function of leg foreword swing may lead to additional progress towards pre-pathological gait.

## Data Availability

The datasets generated during and analysed during the current study are available from the corresponding author on reasonable request.
